# Quadriwave lateral shearing interferometric microscopy with wideband sensitivity enhancement for quantitative phase imaging in real time

**DOI:** 10.1038/s41598-017-00053-7

**Published:** 2017-01-31

**Authors:** Tong Ling, Jiabin Jiang, Rui Zhang, Yongying Yang

**Affiliations:** 1 State Key Laboratory of Modern Optical Instrumentation, College of Optical Science and Engineering, Zhejiang University, Hangzhou, 310027 China; 2 Department of Ophthalmology, Stanford University, CA, 94305 USA

## Abstract

Real-time quantitative phase imaging has tremendous potential in investigating live biological specimens *in vitro*. Here we report on a wideband sensitivity-enhanced interferometric microscopy for quantitative phase imaging in real time by employing two quadriwave lateral shearing interferometers based on randomly encoded hybrid gratings with different lateral shears. Theoretical framework to analyze the measurement sensitivity is firstly proposed, from which the optimal lateral shear pair for sensitivity enhancement is also derived. To accelerate the phase retrieval algorithm for real-time visualization, we develop a fully vectorized path-independent differential leveling phase unwrapping algorithm ready for parallel computing, and the framerate for retrieving the phase from each pair of two 4 mega pixel interferograms is able to reach 47.85 frames per second. Experiment results demonstrate that the wideband sensitivity-enhanced interferometric microscopy is capable of eliminating all the periodical error caused by spectral leaking problem and reducing the temporal standard deviation to the half level compared with phase directly retrieved by the interferogram. Due to its high adaptability, the wideband sensitivity-enhanced interferometric microscopy is promising in retrofitting existing microscopes to quantitative phase microscopes with high measurement precision and real-time visualization.

## Introduction

Quantitative phase microscopy (QPM), which provides additional phase information about the refractive index distribution of transparent specimens, has drawn much attention in both optical and biomedical research. The main advantage of quantitative phase microscopy (QPM) over conventional intensity imaging or fluorescence microscopy is that it is label-free and no dye or fluorescent protein is required to enhance the contrast of the microscopic image, which makes it possible to study live biological specimens *in vitro* ^1–5^.

Ptychography^5–8^ is one of the emerging QPM techniques that can retrieve the phase and amplitude of the scattered wavefield when a coherent illumination beam passes through the specimen under test. The diffraction patterns of the specimen are captured by a CCD camera and the phase retrieval algorithm is carried out based on the extended ptychographic iterative engine (ePIE). Ptychographic microscope can obtain high contrast images free of halo artefact using low magnification microscopic objectives^9, 10^. However, both the acquisition for the tile scan to generate overlapping diffraction patterns and the computation of the ePIE algorithm takes a relatively long time, so it is currently not available to real-time applications. Different from ptychography, the transport-of-intensity equation (TIE) technique employs focus and defocus images to retrieve the phase variation of the wavefront. It can be applied to a conventional intensity microscope directly and the acquisition rate can be set up to 15Hz if an electrically tunable fluidic lens is employed to vary the equivalent focal length of the optical system automatically^11^. By introducing partially coherent light source such as LED to avoid speckle noise, the accuracy and signal-to-noise ratio (SNR) of the TIE technique can be improved and the root-mean-square (RMS) error of the reconstructed phase image will decrease to 12%^12^. Nevertheless, its accuracy still need to be improved if compared with interferometric methods like diffraction phase microscope (DPM)^1, 13–15^, digital holographic microscope (DHM)^16–21^ and the off-axis τ interferometer^22, 23^. For measurement precision, the interferometric technique is now able to reach an optical path stability of about 10 pm with an Adimec camera having larger pixel full well capacity^24^. Meanwhile, to shorten the computation latency, Nvidia’s compute unified device architecture (CUDA) is recently employed in the DHM to provide real-time processing at 34.8 frames per second (fps) for 2048 × 2048 pixel holograms^25^. As a result, with interferometric microscopic techniques, the dynamics of red blood cells (RBCs) was studied^26, 27^ and the isovolumetric cell deformation through membrane electromotility has also been investigated^28^.

Most of the interferometric microscopes, including the DHM, the DPM and the off-axis τ interferometer, are based on Mach-Zehnder interferometry (MZI) or point diffraction interferometry (PDI), in which the interferograms are obtained due to the interference between the reference beam and the sample beam. The precision of these interferometers is of significant relevance to the flatness of the reference wavefront in MZI or the fabrication quality and the actual position and orientation of the pinhole in PDI. Thus meticulous alignment of the optical paths should be carried out carefully to ensure high precision when they will be mounted on an existing microscope. In contrast, without the reference beam, lateral shearing interferometers (LSIs) usually have a more compact structure and a higher tolerance to adapt into other optical systems because of its self-reference feature. Quadriwave lateral shearing interferometers (QWLSIs) are capable of transient phase imaging by acquiring shearing wavefronts in two orthogonal shearing directions in only one single interferogram, and exhibit great suppression over environmental vibration as their optical layouts are designed to be rigorously common-path. The modified Hartmann mask (MHM) for QWLSI consists of a cross grating whose duty ratio is 2/3 and a phase chessboard. The central four orders contains 87% of the output energy^29, 30^, so it is firstly introduced in QWLSI and has been employed for quantitative phase microscopy of living cells as well^31, 32^. However, the interferogram of monochromatic light beams diffracted by the MHM varies periodically as the distance from the MHM to the image plane increases, and the available lateral shear amounts related to this observation distance are also discrete. As the lateral shear amount is closely related to both the dynamic range and the testing sensitivity, a flexible shear amount will be beneficial for the quantitative phase imaging in need of higher sensitivity and precision. In our previous work, a randomly encoded hybrid grating lateral shearing interferometer (REHG-LSI), which is a novel QWLSI based on the randomly encoded hybrid grating (REHG), has been proposed^33^. The REHG consists of a randomly encoded binary amplitude grating and a phase chessboard. The randomly encoded binary amplitude grating simulates the transmittance of the ideal quadriwave grating with tiny encoded masking pixels, while the phase chessboard is a transparent substrate on which the phase modulations of 0 and *π* arrange alternatively. Its Fraunhofer diffractions only contains four orders in the center, which are respectively the +1 and −1 orders in two orthogonal directions. There is no periodical Talbot effect affecting the observation distance^34^ and a continuously variable lateral shear can be obtained with the REHG, which, as a result, makes it possible to control the measurement sensitivity.

**Figure 1:**
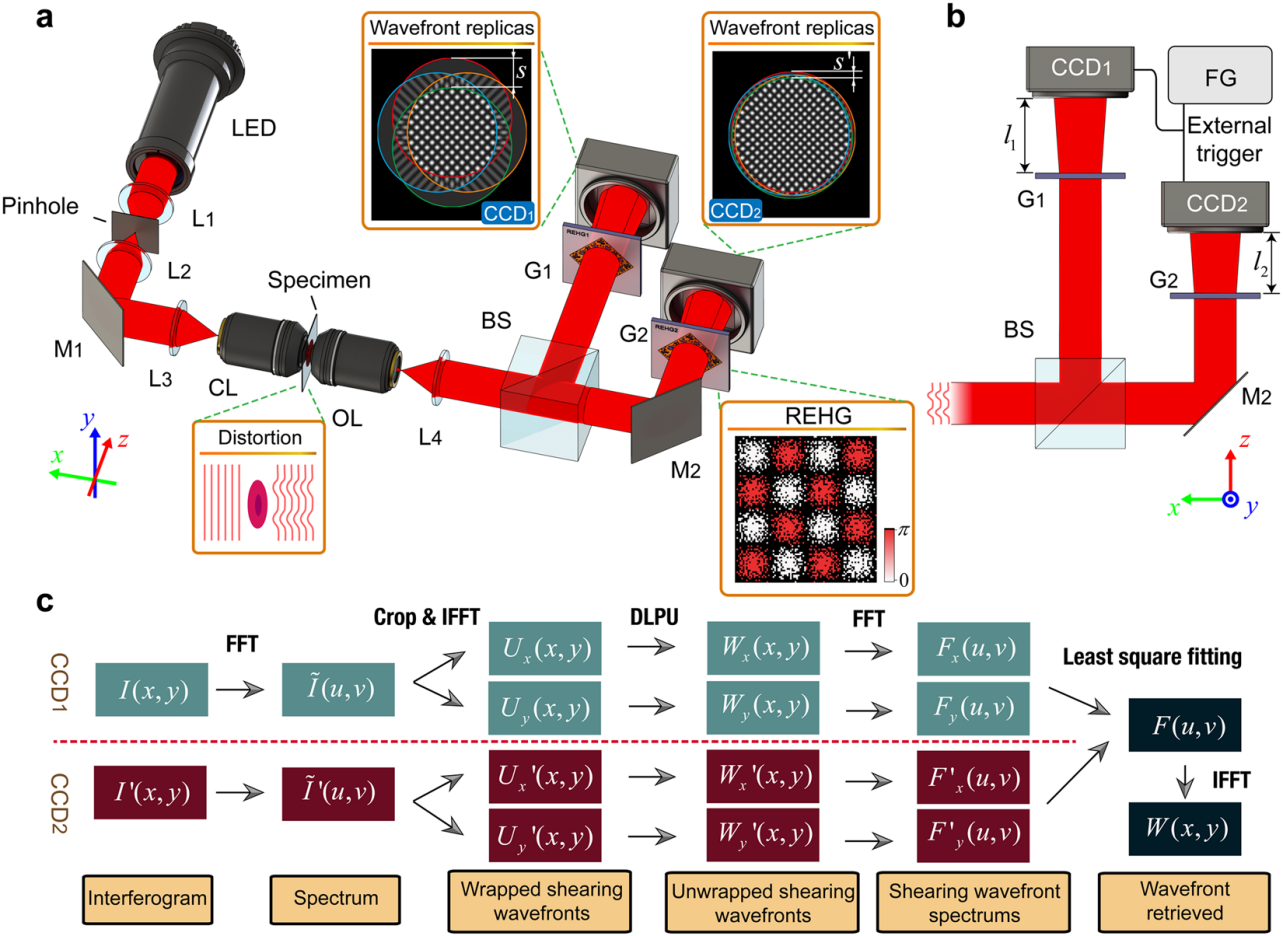
Principle of WSEIM and our phase retrieval algorithm. (**a**) Optical layout of WSEIM: L1, L2, L3 and L4, lenses; M1 and M2, mirrors; CL, condenser lens; OL, objective lens; BS, beam splitter; G1 and G2, REHGs. The light emitted from a LED chip is collimated by a collimator first and filtered by a pinhole to become a plane wave probe when it reaches the specimen. The variation of refractive index distribution in the specimen results in the wavefront distortion of the probe. Then the probe is divided into two beams and each of them is diffracted into four replicas by a REHG. Two quadriwave lateral shearing interferograms with different lateral shear *s* and *s*′ are recorded by CCD1 and CCD2 simultaneously. (**b**) Schematics of the wideband-sensitivity-enhanced phase imaging system: FG, function generator. The difference in the lateral shears is controlled by altering the distances *l*_1_ and *l*_2_ from the REHGs to the CCD image plane. An external trigger signal provided by a function generator is employed to synchronize the image acquisition of two CCDs. (**c**) Schematic diagram of the phase retrieval algorithm for WSEIM. The first three steps are similar with the phase retrieval in the DHM, but the wavefronts obtained with these steps are just the shearing wavefronts. The differential leveling phase unwrapping (DLPU) algorithm is proposed to unwrap the shearing wavefronts. And the wavefront under test will finally be retrieved from its Fourier spectrum, which can be obtained from the four spectrums of shearing wavefronts using least-square fitting method.

In this paper, we describe a novel wideband sensitivity-enhanced interferometric microscopy (WSEIM) based on quadriwave lateral shearing interferometry by employing two REHGs with different lateral shears to enhance its measurement sensitivity and the SNR (Fig. 1). Different from most of the conventional interferometric microscopes which are in need of an ideal reference beam, the WSEIM is self-referenced and only the wavefront signal not the shot noise in quadriwave lateral shearing interferometry is duplicated and sheared to offer shearing wavefronts which may be even larger than the original wavefront signal. Moreover, to obtain the Fourier spectrum of the wavefront under test, the Fourier spectrums of shearing wavefronts in both *x* and *y* directions will be integrated by the least-square fitting method, which again reduces the contribution of stochastic noise to the testing results. The detailed theoretical framework of the wideband sensitivity enhancement is presented in Methods section. To meet the requirements of real-time application, a vectorized differential leveling phase unwrapping (DLPU) algorithm is proposed in this paper and is implemented on Nvidia’s CUDA platform. In fact, although the time complexity of the phase retrieval in WSEIM is more than 4-fold larger than that of the phase retrieval in DHMs, our phase retrieval algorithm for the WSEIM is able to reach a framerate of 47.85 fps by improving the performance of the phase unwrapping algorithm, which usually occupies 50% computation time in conventional phase retrieval algorithm for DHMs on the CPU platform^35^. In addition, due to the rigorously common-path feature of QWLSI, no isolation of environmental vibrations is required for our WSEIM. And the retrofit of an existing microscope from the conventional intensity mode to a WSEIM for phase imaging is also very simple, just by inserting two REHGs in front of the CCD cameras. So the WSEIM is highly adaptable for a plenty of potential applications, including quantitative phase imaging for microfluidic and biomedical research.

## Methods

### Wideband sensitivity-enhanced interferometric microscopy (WSEIM)

WSEIM originates from the quadriwave lateral shearing interferometer (QWLSI) based on the randomly encoded hybrid grating (REHG). As is shown in Fig. 1, a collimator is employed to collect the light emitted from an ordinary LED chip (LR CP7P, SSL 80, OSRAM). The typical dominant wavelength of this LED is 623 nm, and the spectral bandwidth at half maximum intensity is 18 nm. The partially coherent feature of the light source helps mitigate speckle noise resulting from parasitic reflections inside the optics and specimens^12, 13^. Then the light beam is spatially filtered by a combination of a pinhole with a diameter of 100 μm and two lenses L1, L2 to generate a plane wave probe. The refractive index distribution of the specimen will cause wavefront distortion in the plane wave probe, and a 4-*f* telescope system is used to project this wavefront distortion to the image plane. For microscopic imaging of tiny specimens, high-NA condenser lens and objective lens with high magnification are employed in the 4-*f* telescope system; otherwise lenses with relatively large focal lengths can be employed as the condenser lens and the objective lens to offer a wide field of view. The probe is then divided into two beams by a beam splitter. The reflected beam is directly diffracted into four replicas by a REHG G1; but the transmitted beam will impinge on a mirror M2 first, which corrects the mirror effect between the reflected beam and the transmitted beam, and then diffract into four replicas by a second REHG G2. Both of the pitches of these two gratings are designed to be 30 μm to ensure a spatial resolution of 501 × 501 pixels in the final measurement results without the need of zero padding. The quadriwave lateral shearing interferograms formed by interference between the wavefront replicas are recorded by two CCD cameras (BM-500GE, GigE Vision, JAI), whose image planes are placed in the positions conjugated to the object plane where the specimen locates. As the lateral shear between the wavefront replicas on CCD image plane is proportional to the observation distance from the grating to the image plane^36^, two different lateral shears *s* and *s*′ can be achieved by controlling the distances between the gratings and their corresponding CCDs (Fig. 1b).

**Figure 2:**
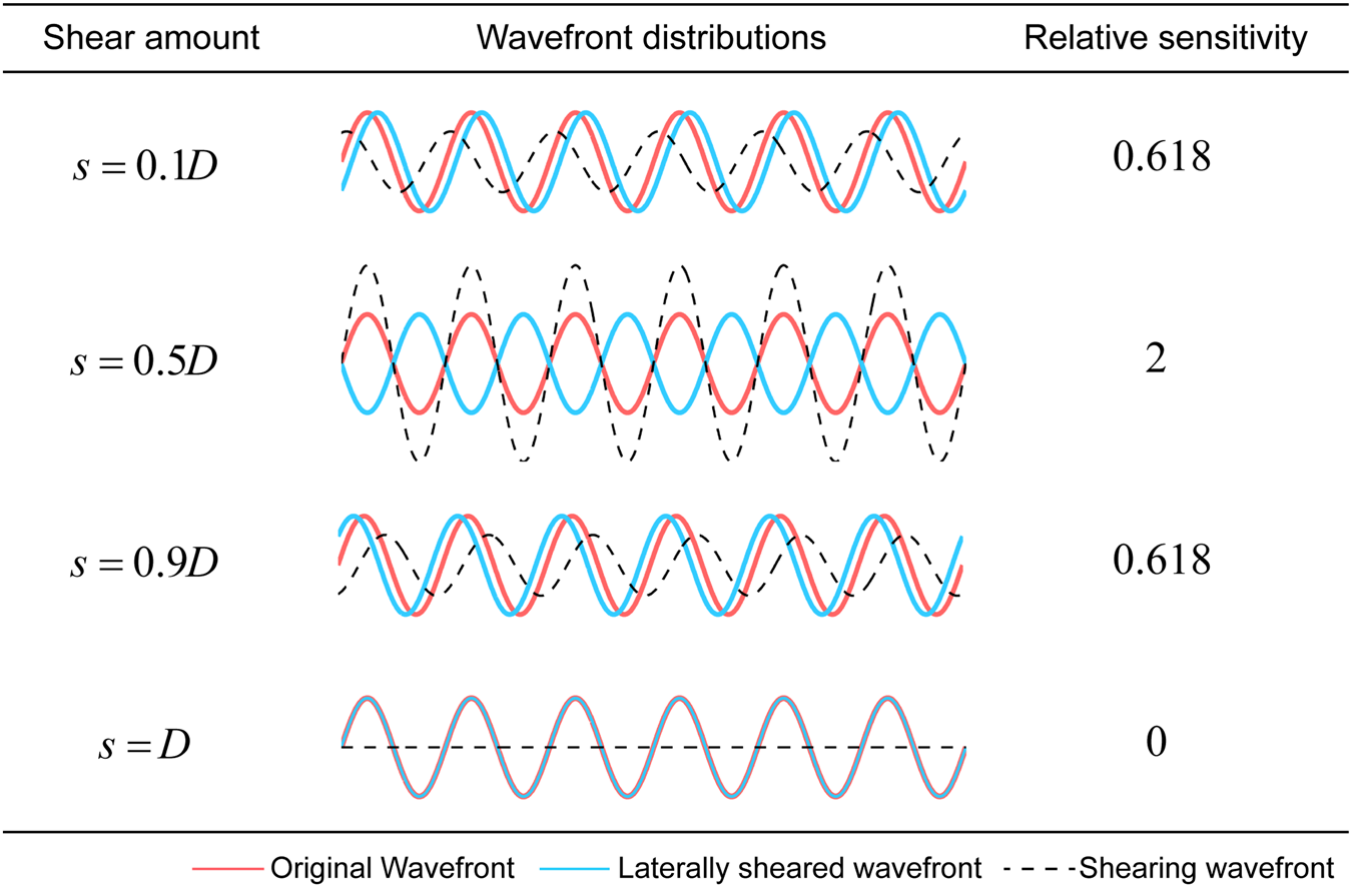
One-dimensional measurement sensitivity of lateral shearing interferometry relative to that of an interferometer with external reference. The relative sensitivity of lateral shearing interferometry ranges from 0 to 2 with the variation of the lateral shear amount when the wavefront under test is in one-dimensional sinusoidal distribution.

In optical metrology, it is generally believed that a large shear amount could help improve the measurement sensitivity and the SNR^37^, as the wavefront aberrations of optical systems mostly concentrate in low spatial frequencies. As a matter of fact, the relationship between the lateral shear amount and the sensitivity is more complicated than simple monotonicity in quantitative phase imaging. Assuming that a wavefront with one-dimensional sinusoidal phase distribution is under test, which is presented in red lines in Fig. 2. The spatial period of the wavefront is *D* and the measurement sensitivity relative to that of a interferometer with external reference like MZI or PDI is *σ*. When the wavefront replica shown in blue is slightly shifted to offer a lateral shear amount of 0.1*D*, the relative sensitivity can only be 0.618. But as the shear amount *s* increases to 0.5*D*, the shearing wavefront which is the difference between the original wavefront and its laterally sheared replica will be amplified and the relative sensitivity can reach its maximum value of 2, which will remarkably improves the signal noise ratio (SNR) and also the measurement precision. For a single sinusoidal wavefront under test, the optimal shear amount for sensitivity enhancement equals half of the period of that wavefront.

Actual wavefronts always contain numerous sinusoidal components with different spatial frequencies. Assume that the wavefront under test is *W*(*x*, *y*), which can be decomposed as follows using inverse Fourier transform,

1 $$W(x,y)={\int }_{-\infty }^{+\infty }{\int }_{-\infty }^{+\infty }F(u,v)\exp [i2\pi (ux+vy)]{\rm{d}}u{\rm{d}}v.$$

By assuming the bandwidth of the sampling system [−Ω, Ω], the wavefront under test *W*(*x*, *y*) can be expressed as an integral of its Fourier spectrum *F*(*u*, *v*) multiplied by an exponential function,

2 $$W(x,y)={\int }_{-{\rm{\Omega }}}^{+{\rm{\Omega }}}{\int }_{-{\rm{\Omega }}}^{+{\rm{\Omega }}}F(u,v)\exp [i2\pi (ux+vy)]{\rm{d}}u{\rm{d}}v.$$

In quadriwave lateral shearing interferometry, the wavefronts measured directly are shearing wavefronts in the *x* and *y* directions *W*_*x*_(*x*, *y*) and *W*_*y*_(*x*, *y*), which are,

3 $${W}_{x}(x,y)=W(x-s/2,y)-W(x+s/2,y),$$

4 $${W}_{y}(x,y)=W(x,y-s/2)-W(x,y+s/2),$$

where *s* is the lateral shear. The Fourier transform of the shearing wavefronts in the *x* and *y* directions *F*_*x*_(*u*, *v*) and *F*_*y*_(*u*, *v*) can be derived from Eqs (2)–(4),

5 $${F}_{x}(u,v)=-F(u,v)\cdot 2i\,\sin (\pi su),$$

6 $${F}_{y}(u,v)=-F(u,v)\cdot 2i\,\sin (\pi sv).$$

Note that some spectrums are amplified in the shearing wavefront spectrums while some decrease even to 0. The Fourier spectrum of the wavefront under test *F*(*u*, *v*) can then be reconstructed using these shearing wavefront spectrums,

7 $$F(u,v)=i\frac{\sin (\pi su){F}_{x}(u,v)+\,\sin (\pi sv){F}_{y}(u,v)}{2[{\sin }^{2}(\pi su)+{\sin }^{2}(\pi sv)]}.$$

Assume that the spectrum uncertainties of the shearing wavefronts in the *x* and *y* directions are Δ_*x*_(*u*, *v*) and Δ_*y*_(*u*, *v*), respectively. The overall uncertainty of the Fourier spectrum of the wavefront to be retrieved is,

8 $${\rm{\Delta }}(u,v)=\frac{\sqrt{{\sin }^{2}(\pi su){\Delta }_{x}^{2}(u,v)+{\sin }^{2}(\pi sv){\Delta }_{y}^{2}(u,v)}}{2[{\sin }^{2}(\pi su)+{\sin }^{2}(\pi sv)]},$$

where *s* is the lateral shear of the QWLSI. Since the shearing wavefronts are measured directly using the same spatial carrier modulation method as the MZI or PDI, their uncertainties are also related to the photon shot noise^24^. The diffraction efficiency of the REHG is 22.17% on its central four orders, and the illumination power should be adjusted so that the maximum intensity of the interferogram captured by CCDs will get close to saturation. If the same type of CCD camera with the same full electron well capacity is employed, the spectrum uncertainties of the shearing wavefronts in the *x* and *y* directions Δ_*x*_(*u*, *v*) and Δ_*y*_(*u*, *v*) will be equal to the uncertainty Δ_0_(*u*, *v*) of a conventional interferometric microscope under the same intensity of interferogram. And the relationship between the uncertainty of a QWLSI Δ(*u*, *v*) and the uncertainty of a conventional interferometric microscope Δ_0_(*u*, *v*) is obtained,

9 $${\rm{\Delta }}(u,v)=\frac{{{\rm{\Delta }}}_{0}(u,v)}{2\sqrt{{\sin }^{2}(\pi su)+{\sin }^{2}(\pi sv)}}.$$

Therefore the sensitivity *σ*(*u*, *v*) of a QWLSI relative to that of a conventional interferometric microscope can be defined as,

10 $$\sigma (u,v)=2\sqrt{{\sin }^{2}(\pi su)+{\sin }^{2}(\pi sv)}.$$

Compared with a conventional interferometric microscope with external reference, the uncertainty of a QWLSI is only $1/2\sqrt{2}$ at the frequencies like,

11 $$u=(2m+1)/2s,\,v=(2n+1)/2s,\quad m,n\in Z.$$

At these frequencies, not the noise but only the signal is amplified by a factor of 2 to achieve higher sensitivity and SNR. However, the sensitivity of the QWLSI goes down again near the following frequencies,

12 $$u=m/s,\,v=n/s,\quad m,n\in Z.$$

Due to the noise in the measured shearing wavefronts, the reduction of sensitivity at these frequencies usually results in a large error in the Fourier spectrum at these frequencies, which is also referred to as spectral leaking problem. This problem can be solved by introducing another lateral shear^38–40^. As is shown in Fig. 1a, if the beam under test is firstly divided by a beam splitter and one more QWLSI interferogram with a different lateral shear *s*′ is obtained, the relative sensitivity *σ*(*u*, *v*) will be obtained as follows taking into consideration the increase of the shot noise by a factor of $\sqrt{2}$,

13 $$\sigma ^{\prime} (u,v)=\sqrt{2}\cdot \sqrt{{\sin }^{2}(\pi su)+{\sin }^{2}(\pi sv)+{\sin }^{2}(\pi s^{\prime} u)+{\sin }^{2}(\pi s^{\prime} v)}.$$

By altering different pairs of lateral shears (*s*, *s*′), the sensitivity performance of the multi-shear QWLSI will also change. To enhance the sensitivity of the multi-shear QWLSI on a wide band of frequencies, the variation of *σ*′(*u*, *v*) should be gentle given the pair of lateral shears, which means the standard deviation of *σ*′(*u*, *v*) in the actual frequency range [−Ω, Ω] × [−Ω, Ω] should be minimized. This criterion and the detailed analysis of the QWLSI sensitivity above lay a theoretical framework in finding an optimal lateral shear pair to achieve wideband sensitivity enhancement.

### Differential leveling phase unwrapping algorithm for real-time applications

**Figure 3:**
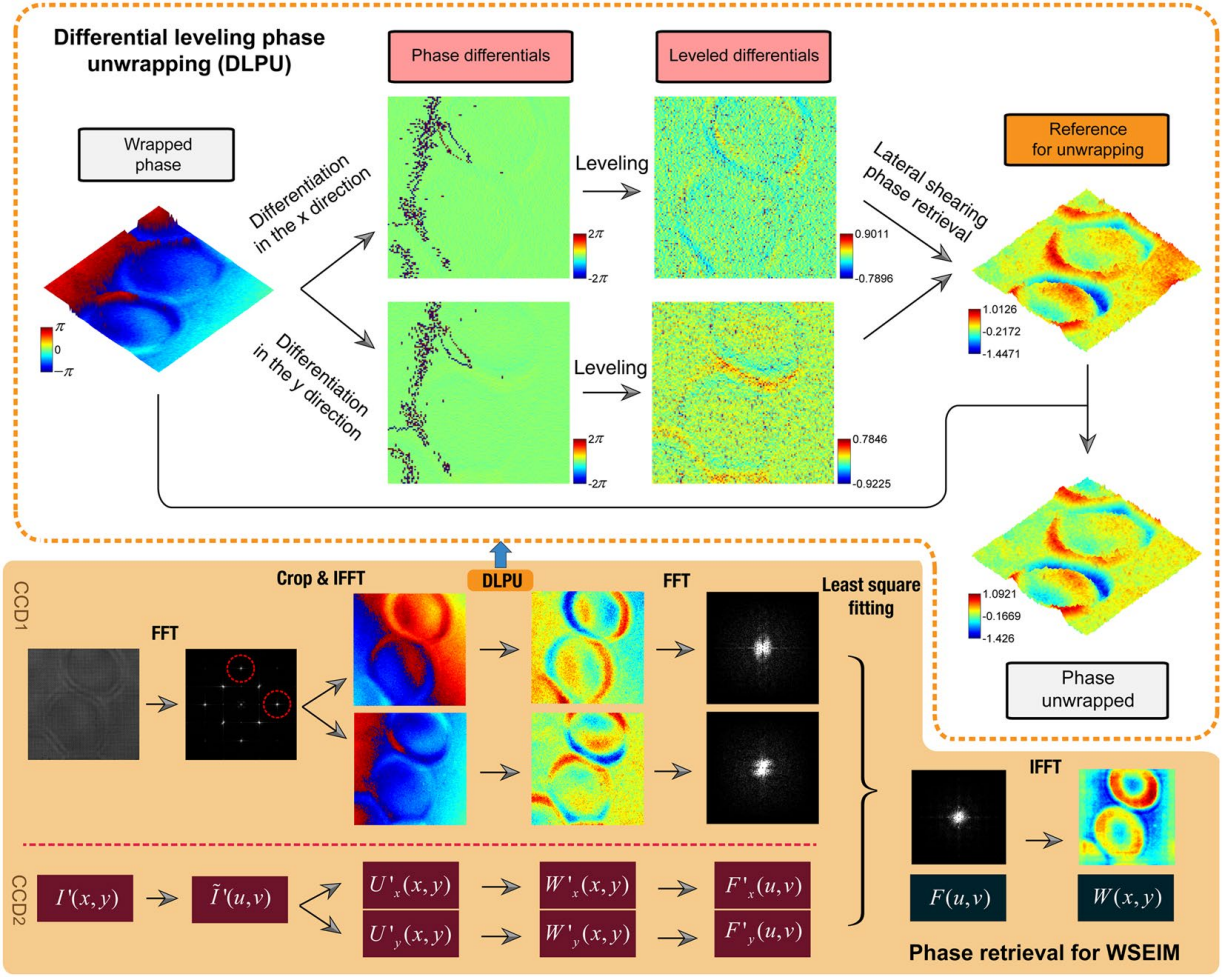
Schematics of the phase retrieval algorithm for WSEIM. The yellow box shows the flowchart of phase retrieval algorithm for WSEIM. The processing procedures in the phase retrieval for WSEIM contain FFTs, IFFTs and the differential leveling phase unwrapping (DLPU) algorithm. In the DLPU shown in the orange dashed box, phase differentials in the *x* and *y* directions are firstly obtained. Then the leveling procedure corrects the ±2*π* jumps in the differentials and a reference phase for unwrapping can be obtained from the leveled differentials by discrete Fourier transform lateral shearing phase retrieval algorithm. The difference in the unit of 2*π* between the wrapped phase and the reference phase determines the number of 2*π* that should be added or subtracted to the wrapped phase at each pixel for obtaining the unwrapped phase.

The phase retrieval algorithm for a QWLSI consists of two steps, the extraction of shearing wavefronts in the *x* and *y* directions from the quadriwave lateral shearing interferogram and the final retrieval of the wavefront under test with these shearing wavefronts^41^. The first step is similar with the phase retrieval in the DPM^14, 42^ and the DHM^15^, but one more wavefront which is the shearing wavefront in the *y* direction needs to be processed in the QWLSI. The WSEIM, which employs two QWLSIs with different lateral shears, is designed to retrieve the wavefront under test with two quadriwave lateral shearing interferograms, and both of them contain two shearing wavefronts. Therefore, the time complexity of the phase retrieval algorithm for WSEIM is at least 4-fold larger than that of the phase retrieval algorithm for DHMs. The detailed phase retrieval steps in Fig. 3 can be generalized as the following three operations, fast Fourier transform (FFT), inverse fast Fourier transform (IFFT) and the phase unwrapping. Among them, both of the FFT and IFFT algorithms with great performance have been developed on Nvidia’s CUDA, but the widely used Goldstein’s branch-cut (GOLD)^15, 43^ and noncontinuous quality-guided path (NCQUAL) phase unwrapping algorithm^44, 45^ can hardly be implemented here by parallel computation because they depend on sequential searching and joining path, though noncontinuous. A robust and fully vectorized path-independent phase unwrapping algorithm with element-wise matrix operations will then help realize all the potentials of the parallel computing system. As a result, the differential leveling phase unwrapping (DLPU) algorithm is proposed to solve this problem.

In the DLPU illustrated in Fig. 3, the wrapped phase is firstly differentiated into the phase differentials in the *x* and *y* directions, respectively. Due to the 2*π* phase jumps in the wrapped phase, some pixels of the differentials will be 2*π* and others will be −2*π*. The next step which is leveling is to restore these pixels to the correct differentials by subtracting or adding 2*π*. Then a phase distribution can be retrieved by the lateral shearing phase retrieval algorithm based on the discrete Fourier transform^46^ using these leveled differentials. To avoid the error produced by this phase retrieval, however, this phase distribution cannot be regarded as the final unwrapped result, but only serves as a reference for the following unwrapping. Tilts are then removed from the wrapped phase and the reference phase, and the difference between these two phases at every pixel determines how many 2*π* jumps the wrapped phase should be added or subtracted at this pixel. The phase unwrapped can finally be obtained by adding or subtracting the corresponding 2*π* jumps at each pixel to the wrapped phase. The lateral shearing phase retrieval in the DLPU only contains FFT, IFFT, adding, element-wise multiplication and division of matrix. Other operations in the DLPU are also independent among the pixels and ready for parallel computing. So the advantage of the DLPU algorithm is that all operations are now vectorized which can be implemented with high performance on a parallel computing platform.

## Results and Discussion

### Wideband sensitivity-enhanced interferometric microscopy (WSEIM)

**Figure 4:**
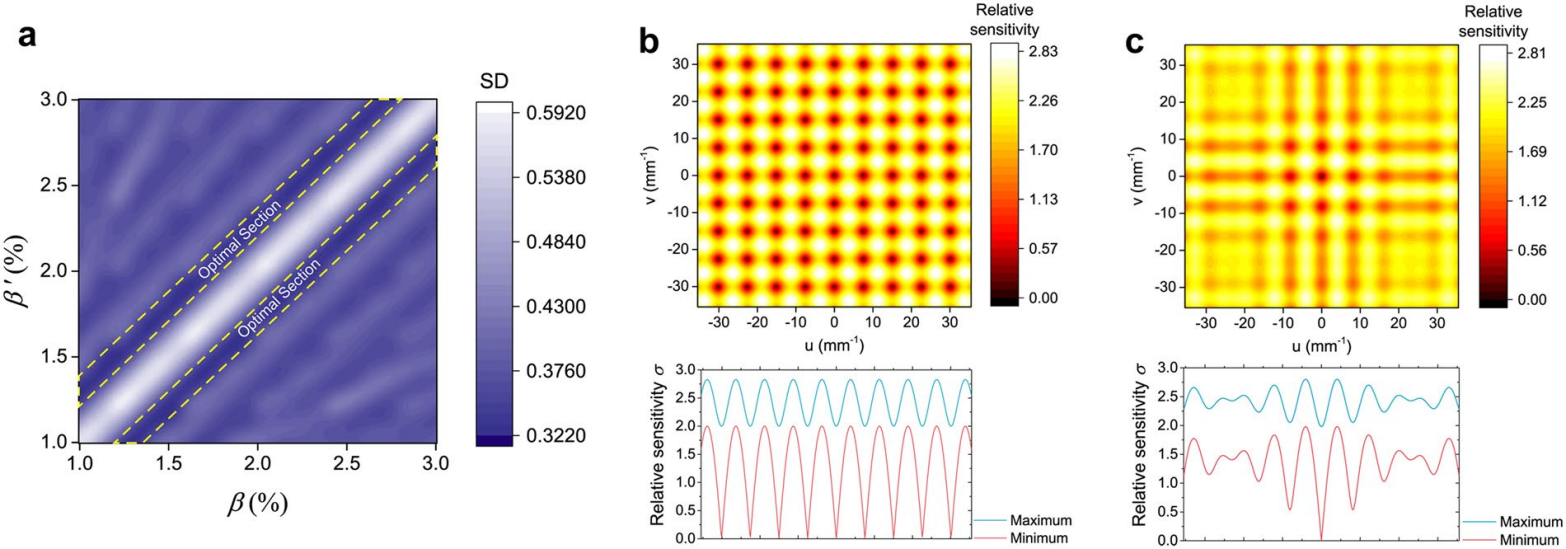
Optimization of lateral shear pair for wideband sensitivity enhancement. (**a**) Map of the standard deviation (SD) of spectral sensitivity distribution when the two lateral shear ratios *β* and *β*′ vary from 1% to 3%. The optimal lateral shear pairs come with low values of SD and are located in the two valleys along each side of the diagonal of the map, which are marked out in this figure. (**b**) Spectral sensitivity distribution when the two lateral shear ratios *β* and *β*′ are both 1.88%. The spatial frequency range was calculated based on the actual image size on the CCD camera and the spatial resolution. The blue and red lines show the maximum and minimum relative sensitivities on each frequencies of *u*, while zero sensitivities are obtained at multiple frequencies. (**c**) Spectral sensitivity distribution when the two lateral shear ratios *β* and *β*′ are 1.88% and 1.57%, respectively. There is only one zero point which is the central point with zero frequency and almost all the spectral relative sensitivities except four points in the center are above 1.0.

For conventional single shear QWLSI, although there are more than one half of the spatial frequencies where the sensitivities relative to the interferometer with external reference are greater than 1, the relative sensitivities can also be as low as zero at some frequencies, as detailed in Methods Section. To enhance the sensitivity on a wide band of spatial frequencies, two quadriwave lateral shearing interferograms with different lateral shears are introduced to complement each other’s zero points on the spectrums. In fact, the optimal pair of lateral shears comes with the equilibrium of the spectral sensitivities, which, in others words, means the standard deviation (SD) of spectral sensitivity distribution should be minimized. As a result, a map of the SD values of spectral sensitivity distribution when the two lateral shear ratio *β* and *β*′, which were respectively the ratios of lateral shear amounts *s* and *s*′ to the size 7.066 mm of the beam incident on the CCD image plane, change from 1% to 3% was firstly obtained (Fig. 4a). The two-dimensional spatial frequencies were calculated using the dimension of 7.066 mm × 7.066 mm and the resolution of 501 × 501 pixels. The diagonal of this map represented the circumstances when the two lateral shears equaled to each other, which could also be regarded as single shear QWLSI. In these cases, the spectral sensitivities were periodical and multiple zero points were located in the sensitivity distribution (Fig. 4b). When the lateral shears went slightly different from each other, there would be a decrease in the SD of spectral sensitivity distribution. In the SD map, two valleys lied along each side of the diagonal peak, and an optimal lateral shear pair could be selected from these two sections. Figure 4c shows the spectral sensitivity distribution when the lateral shear ratio *β* and *β*′ were 1.88% and 1.57%, respectively. Only one point which was the zero frequency exhibited a sensitivity of zero and almost all the spectral relative sensitivities were over 1, compared with the interferometer with external reference. The average spectral relative sensitivity of our WSEIM was around 2, which resulted in the achievement of wideband sensitivity enhancement.

### Real-time phase visualization using WSEIM

Real-time visualization demand that the computation latency should be less than the time interval between every two frames recorded by the camera. For CCDs (BM-500GE, GigE Vision, JAI) employed in the experiment, the framerate of full frame is 15 fps, so the computation latency of phase retrieval should not exceed 66.6 ms. Although the fast and robust phase unwrapping algorithm based on noncontinuous quality-guided path^44^ (NCQUAL) proposed by Herráez *et al.* was employed, the processing of a 2048 × 2048 interferogram pair on CPU (Intel i7-6700H, 2.60 GHz) still took 1417.92 ms (Table 1), which is obviously not available for real-time applications. To accelerate the processing speed, open source ArrayFire library based on CUDA technology which could enable the parallel computing on GPU was introduced. The time cost for the FFT of a single 2048 × 2048 interferogram which was 117.45 ms on CPU had decreased to 1.15 ms on a laptop GPU (GeForce GTX 960 M, 640 CUDA cores), and could further reach 0.49 ms on a high-performance desktop GPU (GeForce GTX Titan Black Edition, 2880 CUDA cores). However, the NCQUAL algorithm for phase unwrapping could hardly be implemented on GPU in parallelism, and it became a handicap for real-time visualization. As a result, a fully vectorized path-independent phase unwrapping algorithm with element-wise matrix operations named differential leveling phase unwrapping (DLPU) algorithm, which is detailed in Methods Section, was proposed and implemented on CUDA platforms. The time cost for phase unwrapping then decreased to 5.54 ms on the GTX 960M and to 3.77 ms on the GTX Titan Black. Moreover, lazy execution (LE) provided by ArrayFire library would automatically construct optimally-sized kernels from the algorithms to be executed. Without the contribution of LE, the total processing time was slightly over the sum of the duration of each step. But when LE was switched on, an even less total processing time could be finally achieved. The phase retrieval framerate of our WSEIM was 24.81 fps on the laptop GTX 960 M and 47.85 fps on the high-end GTX Titan Black, which well met the real-time requirement over the framerate of 15 fps.

**Table 1:** Framerate comparison between different processors.

Duration of steps in sequential order (ms)	Occurrence number	CPU (i7-6700H)	GPU (GTX 960M)	GPU (GTX Titan Black)
FFT of interferogram	×2	117.45	1.15	0.49
Crop & IFFT	×4	21.48	2.33	1.06
Unwrapping	×4	109.93	5.54	3.77
FFT of shearing wavefront	×4	19.21	1.73	0.87
Least square fitting	×1	547.97	7.59	5.12
IFFT of wavefront spectrum	×1	10.78	0.29	0.20
Processing time without LE (ms)		1417.92	49.61	29.15
Processing time with LE (ms)		—	40.31	20.90
Framerate (fps)		0.71	24.81	47.85

The process steps are listed in the sequential order corresponding to the flowchart in Fig. 3, and their occurrence numbers indicate the times each process step occurs in the phase retrieval algorithm. Note that the duration of the second FFT on the 501 × 501 shearing wavefront was a bit larger than that of the first FFT on the 2048 × 2048 interferogram on GPU platforms, due to the fact that the zero padding of 501 × 501 pixels to the size of 2^*N*^ for FFT also consumes time in memory transferring.

### Error analysis and calibration

The error sources of the WSEIM include the wavefront distortion of the probe, the wavelength difference due to the partial coherent light illumination, the translational offset between two cameras and the vibration in the two beams transmitted or reflected by the beam splitter. The wavefront distortion of the probe can be easily eliminated by measuring the background phase image and storing it as the systematic error data for the subtraction in the future measurement. For the wavelength difference, one of the advantages of the ideal QWLSI is that it is theoretically achromatic^30^. Although the difference in the lateral shear amounts of different wavelengths will result in the difference between shearing wavefronts, it will be compensated by the wavelength itself in the phases of these shearing wavefronts, which are exactly what we obtained from a quadriwave lateral shearing interferogram at the first stage. In detail, the wavefront under test *W*(*x*, *y*) is caused by the optical path difference (OPD) of the sample, which is independent of the wavelength. As the lateral shear for QWLSI measurement is usually very small, the shearing wavefronts in Eqs (3) and (4) can be expressed as follows by first-order approximation,

14 $${W}_{x}(x,y)=s\frac{\partial W(x,y)}{\partial x},$$

15 $${W}_{y}(x,y)=s\frac{\partial W(x,y)}{\partial y},$$

where the lateral shear amount *s* can be derived as $2\sqrt{2}l\lambda /d$, *l* is the distance from the grating to the image plane and *d* is the grating pitch. Then the phases of the shearing wavefronts to form and to be retrieved from the interferogram can be obtained as,

16 $${\phi }_{x}(x,y)=\frac{4\pi \sqrt{2}l}{d}\cdot \frac{\partial W(x,y)}{\partial x},$$

17 $${\phi }_{y}(x,y)=\frac{4\pi \sqrt{2}l}{d}\cdot \frac{\partial W(x,y)}{\partial y},$$

which show no dependence on the wavelength actually. For REHG which employs a phase chessboard designed for the specific center wavelength to simulate the 0 and *π* phase steps, unexpected diffraction orders may emerge by broadband wavelength illumination, but as the phase step is only changing from 0.986*π* to 1.014*π* using the red LED chip (LR CP7P, SSL 80, OSRAM) in our WSEIM, the retrieval error caused by this phase step change can also be neglected^34^.

**Figure 5:**
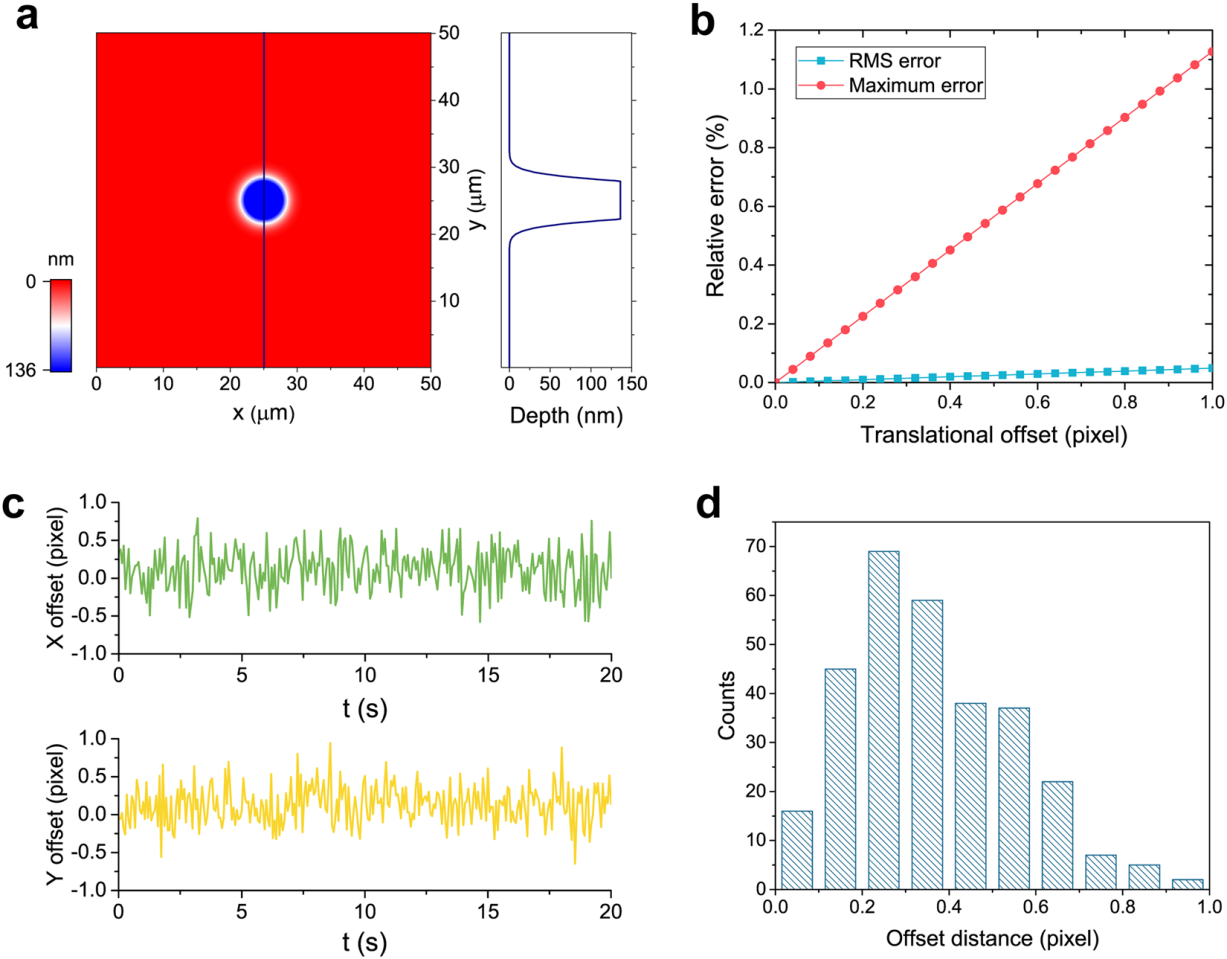
Error analysis of the translational offset and the vibration between the two cameras. (**a**) Modeling of the circular phase target with the diameter of 12 μm and the etching depth of 136 nm on a fused quartz substrate. (**b**) Simulation result of the relative phase retrieval error due to the translational offset between the two cameras. (**c**) Translational offsets in the *x* and *y* directions changing with time due to the environmental vibration in 20 seconds. (**d**) Histogram of the offset distance which is calculated from the translational offsets in the *x* and *y* directions in 20 seconds.

For the analysis of the translational offset and the vibration, another advantage of employing the QWLSIs is that each of the two beams transmitted or reflected by the beam splitter for quadriwave lateral shearing interference is common-path, so either the translational offset or the vibration doesn’t affect the test results at the sensitive interferometric level, all they will influence is the lateral shift of the phase images obtained by the two QWLSIs, which can be also regarded as the same lateral shift of the bright-field images on the two cameras when the REHGs haven’t been set. As a result, the alignment of the two QWLSIs always begins with the alignment of the only two cameras with no REHGs in front, and the translational offset can be reduced by laterally moving one camera on a precise translational stage to match the two bright-field images. However, this error can hardly be fully eliminated when the lateral shift is under one camera pixel. For quantitative analysis, circular phase targets with the same diameter of 12 μm and the same etching depth of 136 nm on a fused quartz substrate in modeling and also in reality were introduced (Fig. 5a). Figure 5b shows the simulation result of the relative phase retrieval error of this phase target due to the translational offset. When the translational offset between the cameras reached one pixel, the maximum error relative to the total range of the phase target was 1.127% and the relative RMS error was 0.049%, and the relationship between these errors and the offset were both linear. Vibration error, considered at the imaging level, can be regarded as the translational offset in the two arms changing with time. By measuring the actual phase target for 20 seconds, the translational offsets in the *x* and *y* directions due to the environmental vibration could be obtained from the centroid displacements of the binarized phase images retrieved from the two QWLSIs (Fig. 5c). The histogram of the offset distance taking both the translational offsets in the *x* and *y* directions into account shows that the largest group of offset distances ranged from 0.2 to 0.4 pixel, and their corresponding phase retrieval errors in Fig. 5b are also acceptable.

### Characterization of etched substrate

**Figure 6:**
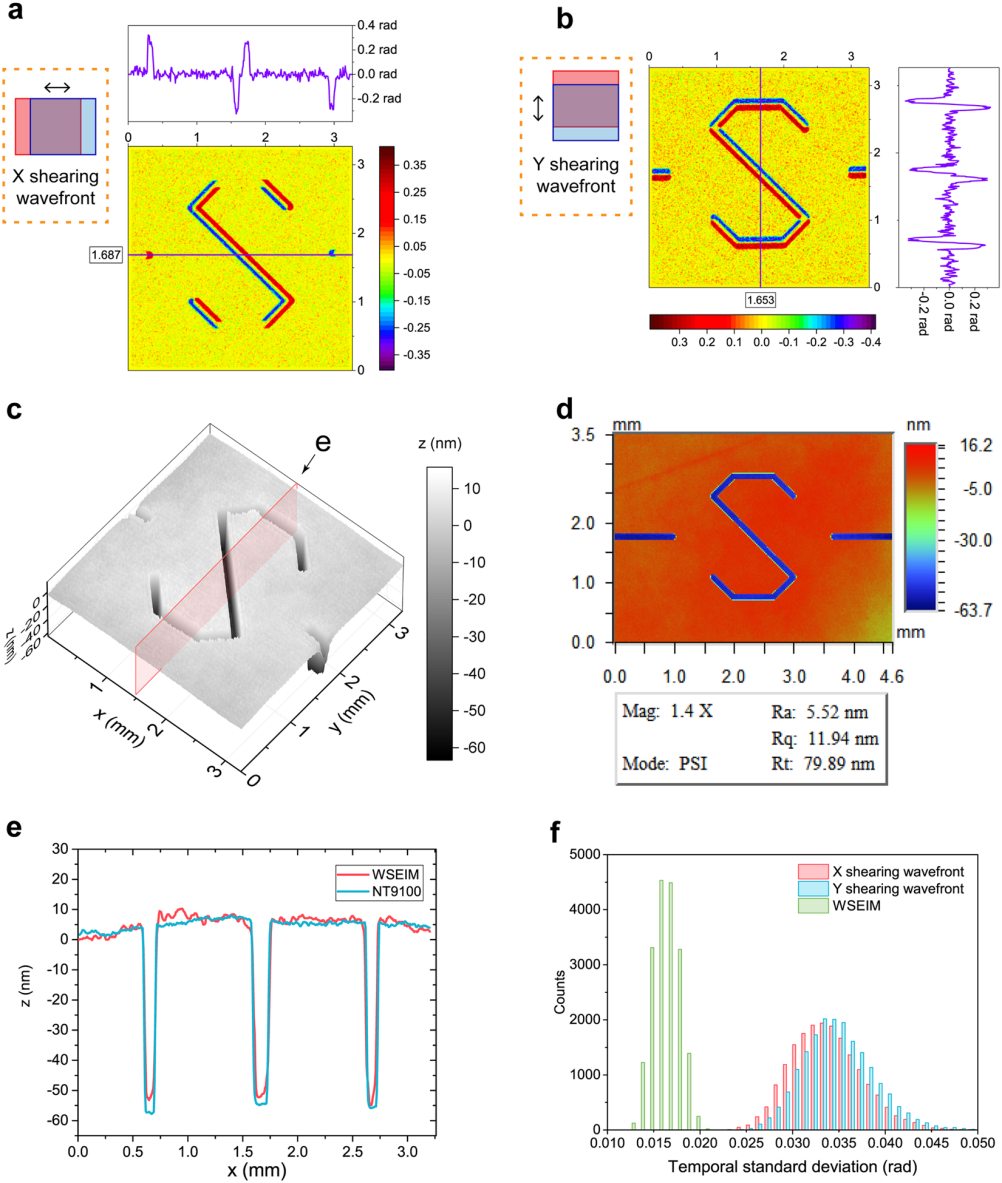
Characterization of etched surface using WSEIM. (**a**) Shearing wavefront in the *x* direction. The purple line on the top shows the one-dimensional distribution along the line *y* = 1.687 mm in the center of the *x* shearing wavefront. (**b**) Shearing wavefront in the *y* direction. The purple line on the right shows the one-dimensional distribution along the line *x* = 1.653 mm in the center of the *y* shearing wavefront. Note that the amplitudes of the shearing wavefronts obtained in (**a**) and (**b**) are both 2-fold larger than that of the original wavefront. (**c**) Profiling result measured by our WSEIM. (**d**) Testing result from the Wyko NT9100 optical profiling system. (**e**) Comparison of the one-dimensional distribution results on the cross section labeled in (**c**) measured by the WSEIM and the Wyko NT9100. (**f**) Histogram of the temporal standard deviation of phase, the shearing wavefronts in the *x* and *y* directions are illustrated in red and blue, while the temporal standard deviation of the wavefront retrieved by WSEIM in green is only half of the temporal standard deviation of the shearing wavefronts.

To demonstrate the precision improvement of our WSEIM, a fused quartz substrate etched with the letter “s” was characterized and the testing results were later compared with the results from a Wyko NT9100 optical profiling system (Fig. 6). As the size of the letter was about 2.5 mm, two aplanatic lenses whose focal lengths are 90 mm were employed as the condenser lens and the objective lens to provide a wide field of view. In the shearing wavefronts in the *x* and *y* directions obtained in Fig. 6a,b, an amplification from the original amplitude of the wavefront signal to its double was observed, which is key to the sensitivity enhancement of WSEIM. The testing results of the etching depth of the letter is shown in Fig. 6c, where the OPD obtained by WSEIM has been converted into the etching depth *z* using the following equation,

18 $$z=\frac{{\rm{OPD}}}{n-1},$$

where *n* is the refractive index of the fused quartz at the wavelength of 623 nm. In comparison with the testing result by the Wyko NT9100 in Fig. 6d, the detailed one-dimensional etching depth distribution in the central cross sections illustrated in Fig. 6c are shown in Fig. 6e. As our WSEIM was designed to measure the wavefront distortion of the fused quartz substrate in transmitted-light configuration, which was different from the direct reflected-light measurement of the etching depth by Wyko NT9100, there existed small differences whose standard deviation is 4.09 nm between the two results. Figure 6f exhibits the histogram of temporal standard deviations of phase which indicate the measurement sensitivities. Due to the relatively low brightness of the LED light filtered by the pinhole, the maximum intensity deviation between every two frames recorded by the CCD was as much as 27 in 8-bit gray scale, while the maximum intensity of the interferogram was only 132 in 8-bit gray scale. As a result, the temporal standard deviation of the shearing wavefronts ranged from 0.024 rad to 0.050 rad with a mean value of 0.034 rad, which also represents the temporal stability of conventional interferometric microscope with external reference employing the same CCD camera under the same intensity of interferogram. In contrast, the temporal standard deviation of the phase results by WSEIM only ranged from 0.013 rad to 0.021 rad, and their mean value of 0.017 rad was only half of the shearing wavefronts’ temporal standard deviation, which agreed well with the theoretical prediction that the average relative sensitivity of the WSEIM is around 2.

### Quantitative phase imaging of the dynamics of RBCs

**Figure 7:**
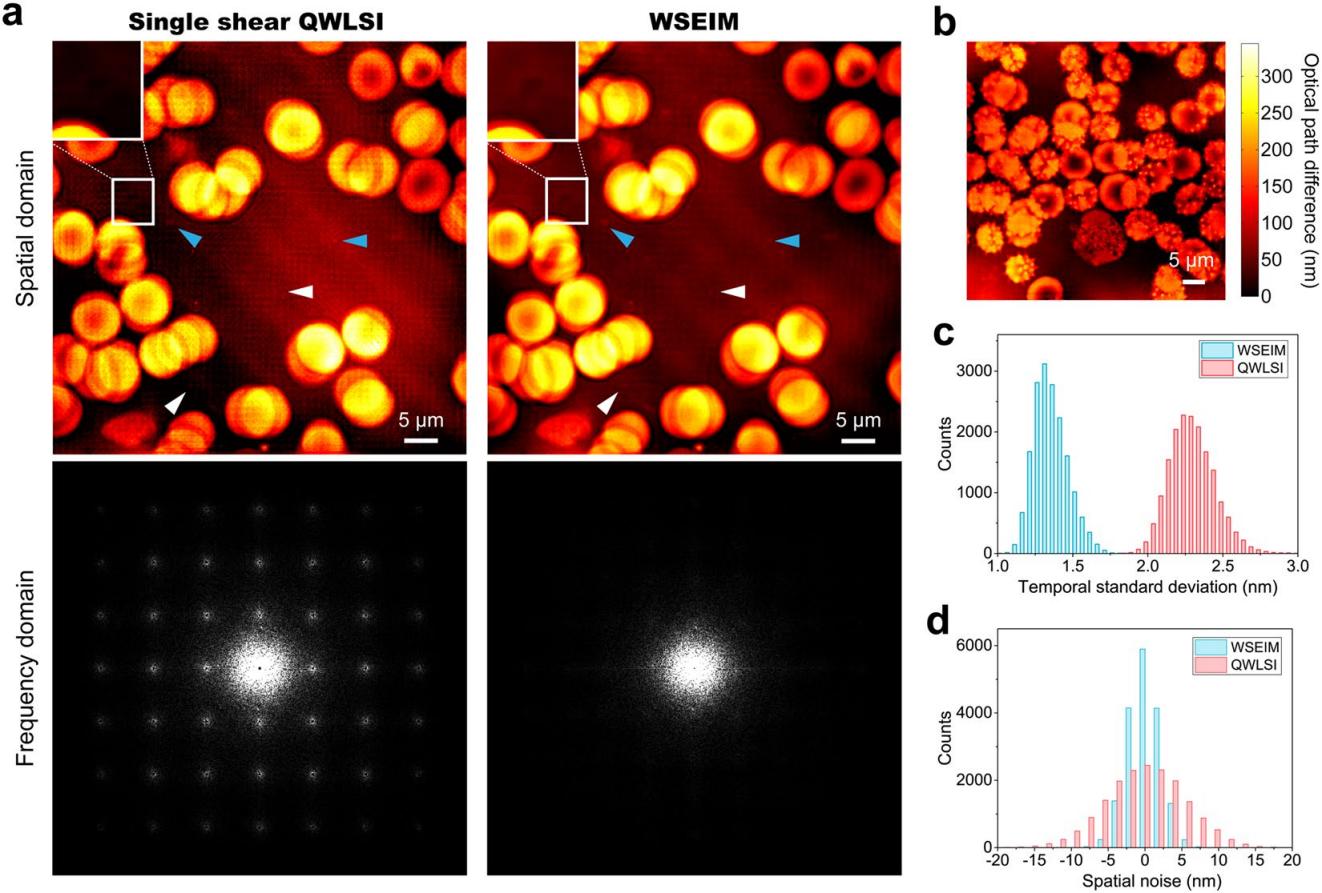
Quantitative phase imaging of RBCs using WSEIM. (**a**) Comparison of the phase imaging results by the single shear QWLSI and the WSEIM. Spatially periodical error emerges in the test result of the single shear QWLSI, as its corresponding Fourier spectrum also has lattice-like errors due to zero measurement sensitivities at these frequencies. Because of wideband sensitivity enhancement, these errors has been eliminated in the test result of the WSEIM. (Insets) A platelet with tiny optical path difference compared with blood plasma is observed by the WSEIM, but it is invisible in the phase image obtained by single shear QWLSI. Blue arrowheads point to the microbes shown in both phase images while white arrowheads point to the microbes only observed in the phase image obtained by the WSEIM. Full time sequence is available as Supplementary video S1. (**b**) Quantitative phase imaging of acanthocytes and a white blood cell to validate the robustness of DLPU algorithm. (**c**) The background standard deviations of the single shear QWLSI and the WSEIM in 20 seconds and (**d**) The spatial noises of the single shear QWLSI and the WSEIM, in which a great improvement in both temporal and spatial SNR can be observed.

**Figure 8:**
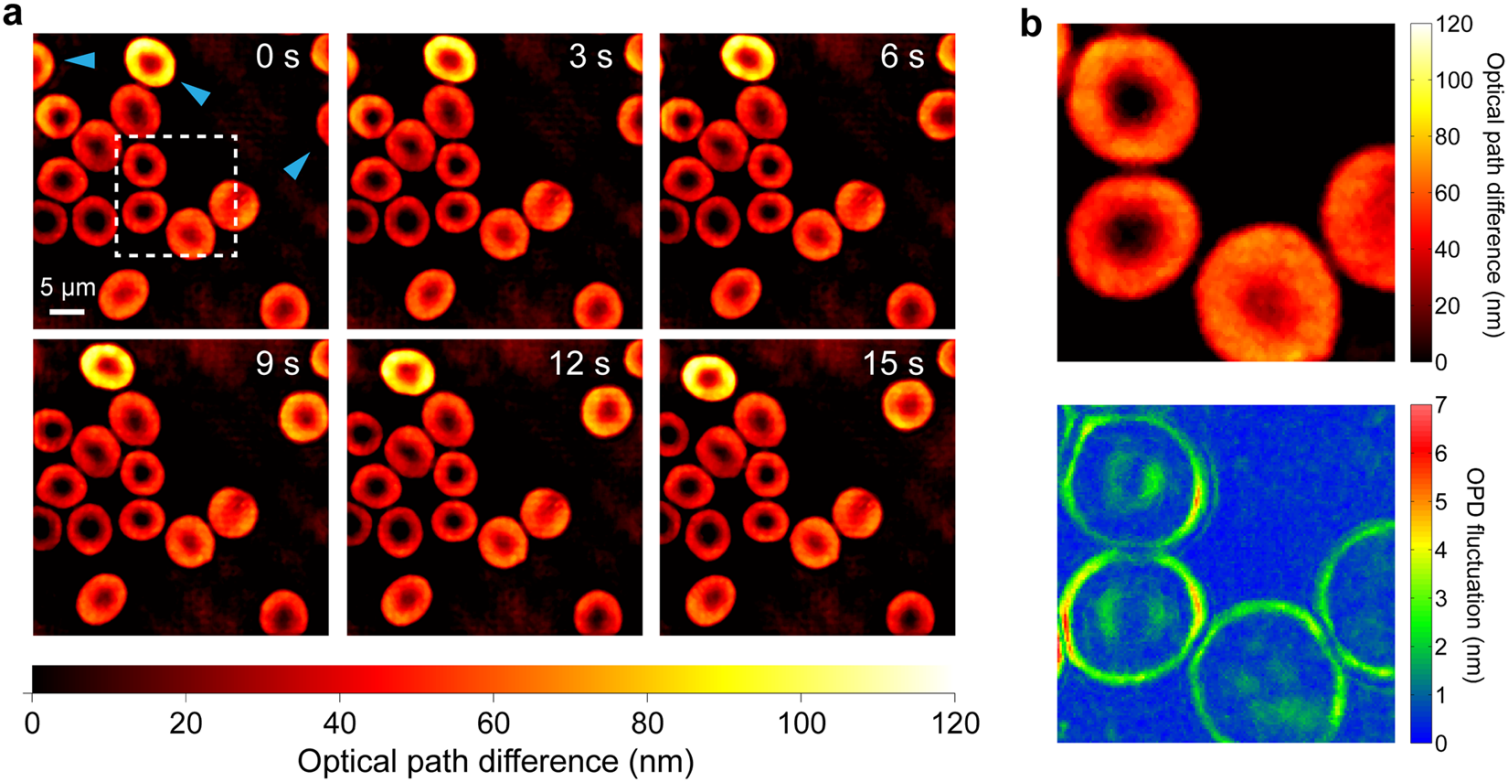
RBC dynamics extracted in real time using WSEIM. (**a**) Time-elapsed phase imaging results of the RBCs flowing in blood plasma in 15 seconds (full time sequence available as Supplementary video S2). Most of the RBCs in the field of view remained in the same position while only those pointed with blue arrowheads were moving. The central region inside the dashed box is zoomed in (**b**), and its OPD standard deviation resulting from the membrane fluctuation of RBCs is also presented below.

The purpose of real-time quantitative phase imaging based on the WSEIM is to observe and study the dynamics of living cells, as a result, samples of sluggishly flowing RBCs sandwiched in two cover glasses were tested by the WSEIM. Two oil-immersed microscopic objectives (UPLSAPO 100X, NA 1.4, Olympus) were employed to project high resolution images onto the CCD cameras through two REHGs. The resolution of the quadriwave lateral shearing interferograms was 2048 × 2048 pixels. The distances from the REHGs to the CCD image planes were adjusted so that the two lateral shear ratios *β* and *β*′ were set to 1.93% and 1.55%, respectively. This lateral shear pair were selected in the optimal section and was able to enhance the sensitivity on a wide band of frequencies. Comparison of the phase imaging results obtained by the WSEIM and conventional single shear QWLSI is shown in Fig. 7a. Due to the zero sensitivities at multiple frequencies, the SNR went down and the noise was then amplified by the small denominator in Eq. (7) around these frequencies, which resulted in a lattice-like error emerged in the Fourier spectrum of the phase image obtained by single shear QWLSI, and also the corresponding periodical error in the spatial domain. In comparison, these errors caused by spectral leaking problem were eliminated in the testing result of the WSEIM, and the temporal standard deviation of background and the spatial noise were also greatly reduced (Fig. 7c,d). Moreover, one platelet and two microbes which were invisible by the single shear QWLSI could then be observed by the WSEIM. Clusters of acanthocytes and white blood cells, which contained more information on higher frequencies, were also imaged by the WSEIM to validate the robustness of our real-time phase retrieval algorithm, especially the performance of the DLPU algorithm (Fig. 7b). No obvious discontinuity was seen in the phase image and details was well presented in a resolution of 501 × 501 pixels without zero padding. Figure 8 shows the time-elapsed dynamic measurement results of the RBCs flowing in blood plasma in 15 seconds. To study the membrane fluctuation of the RBCs in the dashed box in Fig. 8a, their OPD fluctuation in a time interval of 3 seconds was measured as well (Fig. 8b).

## Conclusion

We have presented wideband sensitivity-enhanced interferometric microscopy (WSEIM) based on quadriwave lateral shearing interferometry (QWLSI) for real-time quantitative phase imaging. Theoretical framework that calculate the spectral relative sensitivities of a QWLSI is firstly proposed by introducing Fourier analysis into resolving the relationship between the shearing wavefronts and the original wavefront signal, and the sensitivity enhancement on a wide band of spatial frequencies can be achieved with the optimal selection of the lateral shear pair, which comes with the minimum standard deviation of spectral relative sensitivity. Agreeing with the theoretical prediction, the WSEIM, which combines two QWLSIs with the optimal lateral shear pair together, can successfully reduce the temporal standard deviation to its half and eliminate all the periodical error caused by spectral leaking problem.

To achieve the goal of real-time visualization, a fully vectorized phase retrieval algorithm, including the novel differential leveling phase unwrapping (DLPU) algorithm, is developed for the computation on parallel computing platforms. Utilizing the similar lateral shearing phase retrieval algorithm with a lateral shear of one pixel instead in the DLPU algorithm, no unwrapping path is ever needed and the processes will be only FFT, IFFT and other matrix operations, which have already been optimized by the Nvidia’s CUDA community. Therefore, our phase retrieval algorithm for the WSEIM is able to reach a framerate of 24.81 fps on a laptop GPU and a framerate of 47.85 fps on a high-end desktop GPU. As the first three steps of our phase retrieval algorithm in WSEIM is similar to those of the DHMs, it will also benefit the acceleration of the phase retrieval process in conventional interferometric microscopes with external reference.

The WSEIM with real-time visualization provides an easy access to retrofitting existing bright-field microscopes into quantitative phase microscopes just by inserting two randomly encoded hybrid gratings (REHGs) in front of the CCD image planes. No scanning system for phase shifting is required and the WSEIM can be implemented without any isolation of environmental vibration, as its optical path for quadriwave lateral shearing interference is rigorously common-path. Thanks to the sensitivity enhancement of WSEIM, a temporal OPD standard deviation of 1.32 nm can also be obtained even though the largest deviation between every two frames recorded by the CCD is as much as 20.5% relative to the maximum intensity due to the lack of photons. If the brightness can be further increased using a short-coherence solid-state laser source, such as the Ti:Sapphire laser^47^, and CCDs with larger pixel full well capacity^24^ are going to be employed at the same time, the WSEIM will then fully realize its potentials in sensitivity enhancement and offer the opportunity of new discoveries through real-time observation of the phase variations and fluctuations of living cells.

## Electronic supplementary material

Supplementary Information

Supplementary video S1

Supplementary video S2

## References

[1] Popescu G, Ikeda T, Dasari RR, Feld MS (2006). Diffraction phase microscopy for quantifying cell structure and dynamics. Opt. Lett..

[2] Chen CL (2016). Deep Learning in Label-free Cell Classification. Sci. Rep..

[3] Sugiyama N (2012). Label-free characterization of living human induced pluripotent stem cells by subcellular topographic imaging technique using full-field quantitative phase microscopy coupled with interference reflection microscopy. Biomed. Opt. Express.

[4] Shaked NT, Satterwhite LL, Bursac N, Wax A (2010). Whole-cell-analysis of live cardiomyocytes using wide-field interferometric phase microscopy. Biomed. Opt. Express.

[5] Marrison J, Räty L, Marriott P, O’Toole P (2013). Ptychography–a label free, high-contrast imaging technique for live cells using quantitative phase information. Sci. Rep..

[6] Humphry MJ, Kraus B, Hurst AC, Maiden AM, Rodenburg JM (2012). Ptychographic electron microscopy using high-angle dark-field scattering for sub-nanometre resolution imaging. Nat. Commun..

[7] Thibault P, Menzel A (2013). Reconstructing state mixtures from diffraction measurements. Nature.

[8] Suman R (2016). Label-free imaging to study phenotypic behavioural traits of cells in complex co-cultures. Sci. Rep..

[9] Ou X, Horstmeyer R, Yang C, Zheng G (2013). Quantitative phase imaging via Fourier ptychographic microscopy. Opt. Lett..

[10] Ou X, Horstmeyer R, Zheng G, Yang C (2015). High numerical aperture Fourier ptychography: principle, implementation and characterization. Opt. Express.

[11] Zuo C, Chen Q, Qu W, Asundi A (2013). High-speed transport-of-intensity phase microscopy with an electrically tunable lens. Opt. Express.

[12] Rodrigo JA, Alieva T (2014). Rapid quantitative phase imaging for partially coherent light microscopy. Opt. Express.

[13] Nguyen ,TH, Edwards C, Goddard LL, Popescu ,G (2014). Quantitative phase imaging with partially coherent illumination. Opt. Lett..

[14] Pham HV, Edwards C, Goddard LL, Popescu G (2013). Fast phase reconstruction in white light diffraction phase microscopy. Appl. Opt..

[15] Bhaduri B (2014). Diffraction phase microscopy: principles and applications in materials and life sciences. Adv. Opt. Photon..

[16] Cuche E, Bevilacqua F, Depeursinge C (1999). Digital holography for quantitative phase-contrast imaging. Opt. Lett..

[17] Carl D, Kemper B, Wernicke G, von Bally G (2004). Parameter-optimized digital holographic microscope for high-resolution living-cell analysis. Appl. Opt..

[18] Mann CJ, Yu L, Lo C-M, Kim MK (2005). High-resolution quantitative phase-contrast microscopy by digital holography. Opt. Express.

[19] Paturzo M (2008). Super-resolution in digital holography by a two-dimensional dynamic phase grating. Opt. Express.

[20] Weijuan Q, Yingjie Y, Choo CO, Asundi A (2009). Digital holographic microscopy with physical phase compensation. Opt. Lett..

[21] Tsang PWM, Cheung KWK, Kim T, Kim YS, Poon TC (2011). Fast reconstruction of sectional images in digital holography. Opt. Lett..

[22] Girshovitz P, Shaked NT (2013). Compact and portable low-coherence interferometer with off-axis geometry for quantitative phase microscopy and nanoscopy. Opt. Express.

[23] Girshovitz P, Shaked NT (2014). Doubling the field of view in off-axis low-coherence interferometric imaging. Light-Sci. Appl.

[24] Hosseini P (2016). Pushing phase and amplitude sensitivity limits in interferometric microscopy. Opt. Lett..

[25] Backoach O, Kariv S, Girshovitz P, Shaked NT (2016). Fast phase processing in off-axis holography by CUDA including parallel phase unwrapping. Opt. Express.

[26] Kim Y (2014). Common-path diffraction optical tomography for investigation of three-dimensional structures and dynamics of biological cells. Opt. Express.

[27] Kim, Y. et al. Profiling individual human red blood cells using common-path diffraction optical tomography. *Sci. Rep.* **4**, doi:10.1038/srep06659 (2014). 10.1038/srep06659 PMC4200412 25322756

[28] Oh S (2012). Label-Free Imaging of Membrane Potential Using Membrane Electromotility. Biophys. J..

[29] Primot J, Guérineau N (2000). Extended Hartmann Test Based on the Pseudoguiding Property of a Hartmann Mask Completed by a Phase Chessboard. Appl. Opt..

[30] Chanteloup JC (2005). Multiple-wave lateral shearing interferometry for wave-front sensing. Appl. Opt..

[31] Bon P, Maucort G, Wattellier B, Monneret S (2009). Quadriwave lateral shearing interferometry for quantitative phase microscopy of living cells. Opt. Express.

[32] Bon, P. *et al.* Three-dimensional nanometre localization of nanoparticles to enhance super-resolution microscopy. *Nat. Commun.* **6**, doi:10.1038/ncomms8764 (2015). 10.1038/ncomms8764 PMC4525210 26212705

[33] Ling T (2015). Quadriwave lateral shearing interferometer based on a randomly encoded hybrid grating. Opt. Lett..

[34] Li J, Tang F, Wang X, Dai F, Zhang H (2015). Analysis of lateral shearing interferometry without self-imaging limitations. Appl. Opt..

[35] Girshovitz P, Shaked NT (2015). Fast phase processing in off-axis holography using multiplexing with complex encoding and live-cell fluctuation map calculation in real-time. Opt. Express.

[36] Ling T (2015). General measurement of optical system aberrations with a continuously variable lateral shear ratio by a randomly encoded hybrid grating. Appl. Opt..

[37] Malacara, D. *Optical Shop Testing*. 126 (John Wiley & Sons, 2007).

[38] Elster C, Weingärtner I (1999). Exact wave-front reconstruction from two lateral shearing interferograms. J. Opt. Soc. Am. A.

[39] Elster C (2000). Exact two-dimensional wave-front reconstruction from lateral shearing interferograms with large shears. Appl. Opt..

[40] Guo Y-f, Chen H, Xu J, Ding J (2012). Two-dimensional wavefront reconstruction from lateral multi-shear interferograms. Opt. Express.

[41] Ling T, Yang Y, Liu D, Yue X, Jiang J (2015). Retrieval of phase distributions from the quadriwave lateral shearing interferogram obtained by randomly encoded hybrid grating. Proc. SPIE.

[42] Takeda M, Ina H, Kobayashi S (1982). Fourier-transform method of fringe-pattern analysis for computer-based topography and interferometry. J. Opt. Soc. Am..

[43] Goldstein RM, Zebker HA, Werner CL (1988). Satellite radar interferometry: Two-dimensional phase unwrapping. Radio Sci..

[44] Herráez MA, Burton DR, Lalor MJ, Gdeisat MA (2002). Fast two-dimensional phase-unwrapping algorithm based on sorting by reliability following a noncontinuous path. Appl. Opt..

[45] Parkhurst J, Price G, Sharrock P, Moore C (2011). Phase unwrapping algorithms for use in a true real-time optical body sensor system for use during radiotherapy. Appl. Opt..

[46] Dubra A, Paterson C, Dainty C (2004). Wave-front reconstruction from shear phase maps by use of the discrete Fourier transform. Appl. Opt..

[47] Witte S (2012). Short-coherence off-axis holographic phase microscopy of live cell dynamics. Biomed. Opt. Express.

