# Supplementary material for: Quadriwave lateral shearing interferometric microscopy with wideband sensitivity enhancement for quantitative phase imaging in real time
Source: Sci Rep. 2017 Jan 31;7:9. doi: 10.1038/s41598-017-00053-7 (PMC5428360; doi:10.1038/s41598-017-00053-7)
Supplement: Supplementary file 1 — Supplementary Information [file 41598_2017_53_MOESM1_ESM.doc]

**Quadriwave lateral shearing interferometric microscopy with wideband sensitivity enhancement for quantitative phase imaging in real time**

# Tong Ling^†^, Jiabin Jiang, Rui Zhang, and Yongying Yang^*^

State Key Laboratory of Modern Optical Instrumentation, College of Optical Science and Engineering, Zhejiang University, Hangzhou, 310027, China.

*[yyyang07@163.com](mailto:yyyang07@163.com)

†[tongling@stanford.edu](mailto:tongling@stanford.edu), Present address: Department of Ophthalmology, Stanford University, CA, 94305, USA.

# VIDEO LEGENDS

**Supplementary Video S1. Observation of RBCs flowing with microbes moving.** To avoid acanthocytosis in protoplasmic blood, we employed ultrasonic cleaner instead of alcohol disinfectant to clean the two cover glasses, which gave rise to the emergence of some microbes with their size around $500\text{ μm}$, but also gave us a chance to observe the active movement of these tiny specimens without extrogenous markers.

**Supplementary Video S2. Observation of RBCs sluggishly flowing by WSEIM.** Fluctuation of the RBCs’ membrane can be observed from this video dynamically.
